# Urgent right ventricular outflow tract stenting in a dual risks of thrombosis and bleeding in uncorrected adult with cyanotic congenital heart disease: a case report

**DOI:** 10.3389/fcvm.2026.1819863

**Published:** 2026-07-02

**Authors:** Hiradipta Ardining, Brian Mendel, Radityo Prakoso, Bambang Widyantoro

**Affiliations:** Department of Cardiology and Vascular Medicine, Faculty of Medicine, Universitas Indonesia–National Cardiovascular Center Harapan Kita, Jakarta, Indonesia

**Keywords:** adult congenital heart disease (ACHD), bleeding risk, cyanotic heart disease, RVOT stenting, thrombosis

## Abstract

**Background:**

Uncorrected adults congenital heart disease (ACHD) may present with profound hypoxia and dual risks of thrombosis and bleeding, posing major challenges for definitive surgical repair. In such settings, right ventricular outflow tract (RVOT) stenting may provide a life-saving palliative option.

**Case presentation:**

We report a 30-year-old woman with double-outlet right ventricle, subaortic ventricular septal defect, and severe infundibular pulmonary stenosis who suffered cardiac arrest due to profound hypoxia. Her clinical profile was complicated by polycythemia, upper extremity deep vein thrombosis, and active gastrointestinal bleeding. In light of prohibitive surgical risk, urgent RVOT stenting was undertaken, requiring multiple technical adaptations due to severe obstruction. Post-procedure, oxygen saturation improved to 92%, gastrointestinal bleeding ceased, and deep vein thrombosis regressed without anticoagulation. She subsequently completed phase II cardiac rehabilitation with improved functional capacity.

**Conclusion:**

Urgent RVOT stenting can serve as a feasible therapeutic option in high-risk ACHD patients, effectively restoring pulmonary blood flow while simultaneously stabilizing thrombotic and bleeding complications.

## Background

With advancements in recent decades, management of congenital heart diseases (CHD) have improved, with 97% of patients surviving into adulthood. However, some uncorrected ACHD present because of delayed diagnosis, leading to more severe clinical presentations. Chronic hypoxia may result in increased erythropoiesis and hyperviscocity, putting them at risk of thrombosis. In contrast, these patients may also have a heightened bleeding risk because of thrombocytopenia, platelet dysfunction, and clotting factor deficiencies (1–4).

Fallot-like physiology, including tetralogy of Fallot (TOF) and Fallot-type double outlet right ventricle (DORV) with right ventricular outflow tract obstruction (RVOTO) and ventricular septal defect (VSD), represents one of the most common forms of cyanotic congenital heart disease worldwide. RVOT stenting is one of the palliative procedures of choice, particularly for patients who are yet to be suitable for surgical repair. These practices are usually performed in patients with refractory hypoxic spells and are considered lifesaving through significantly improving pulmonary blood flow and oxygen saturation in cases of severe pulmonary under perfusion. Even though RVOT stenting is considered safe and effective, concerns remain regarding patient with high risk of thrombosis and bleeding risk (5–8).

This report presents a complex case of 30-year-old woman with Fallot-type double outlet right ventricle (DORV), subaortic ventricular septal defect (VSD), and severe pulmonary stenosis (PS) who underwent RVOT stenting with risks of thrombosis and bleeding.

## Case presentation

A 30-year-old female presented to the emergency department with a chief complaint of seizures that began 30 min prior to admission. The seizures were generalized, occurring three times in total with each episode lasting less than five minutes. The seizures were preceded by a one-day history of watery diarrhea and fever. Additionally, patient also reported swelling of the left arm for the past month. No history of recent immobilization, trauma to the limb, generalized edema, malignancy, or known hematologic disorders was noted. There was no bleeding on admission. The patient had a previous seizure a few months prior, which has resolved spontaneously, and was not on any antiepileptic medications. The patient was previously diagnosed with DORV, subaortic VSD, and severe infundibular PS. Her medical history also included deep vein thrombosis (DVT) involving the left subclavian, axillary, brachial, and cephalic veins; polycythemia vera; bicytopenia; bilateral pleural effusion; and a complicated urinary tract infection (UTI).

The patient has been cyanotic since the age of five months. She was recommended to undergo surgical intervention at ten months of age but did not proceed due to financial issues. She continued regular follow-up at the outpatient clinic until high school but did not pursue further medical care thereafter. At the age of 28, her hypoxic spell worsened. No other significant medical history was noted.

On admission, the patient presented with a delirious level of consciousness, with a Glasgow Coma Scale (GCS) score of 10/15 (E2V3M5). Her vital signs showed a blood pressure of 84/50 mmHg, heart rate of 78 bpm, respiratory rate of 18x/minutes, and peripheral oxygen saturation of 78%. Her BMI was 17.1 kg/m^2^. Physical examination revealed elevated jugular venous pressure (JVP) and a grade 3/6 ejection systolic murmur at the upper left sternal border, with no gallop. Additionally, she had ascites, and clubbing finger was noted alongside edema of the left upper extremity and bilateral lower extremities. Laboratory investigations showed polycythemia (Hb 20.0), leukocytosis (Leu 10.390), thrombocytopenia (102.000), hyponatremia (Na 129), and hyperkalemia (K 5.8). Baseline coagulation profile showed INR 2.23 and PT 22.2 s. Arterial blood gas analysis revealed metabolic acidosis (pH 7.39, pCO2 27.9, HCO3 17.1, BE −8.1). Bedside echocardiography demonstrated DORV of Fallot type, with severe infundibular PS and subaortic VSD. Duplex ultrasound of the upper extremity revealed thrombus in the left subclavian, axillary, brachial, and cephalic veins. Arterial flow in both upper extremities was normal. Evidence of deep vein thrombosis (DVT) was found on lower extremity ultrasound.

Her daily medication was Propranolol 20 mg t.i.d, Rivaroxaban 10 mg OD, and Clopidogrel 75 mg OD. The patient was admitted to the intermediate care ward with a plan for elective RVOT stenting. On the third day of admission, the patient experienced severe hypoxic spell followed by cardiac arrest. She was successfully resuscitated with return of spontaneous circulation (ROSC), intubated, and transferred to the cardiovascular care unit (CVCU). An urgent right ventricular outflow tract (RVOT) stenting was then escalated. The patient was transferred to the catheterization laboratory while already intubated and mechanically ventilated, with the procedure performed under general anesthesia and invasive monitoring. Procedural sedation was maintained with intravenous fentanyl 12.5 mcg/hour and midazolam 3 mg/hour. Pre-procedural echocardiography evaluation revealed: DORV Fallot type, very tight infundibular PS, annulus pulmonary valve diameter 15 mm, infundibular length 15 mm, infundibular diameter 3 mm measured in diastole, no pericardial effusion. LV and RV function were decreased and the RVOT was very tight with nearly no PA flow. The presence of tricuspid regurgitation, dilated right-sided chambers, and RV dysfunction contributed to the technical difficulty of RVOT entry and device delivery.

**Figure 1:**
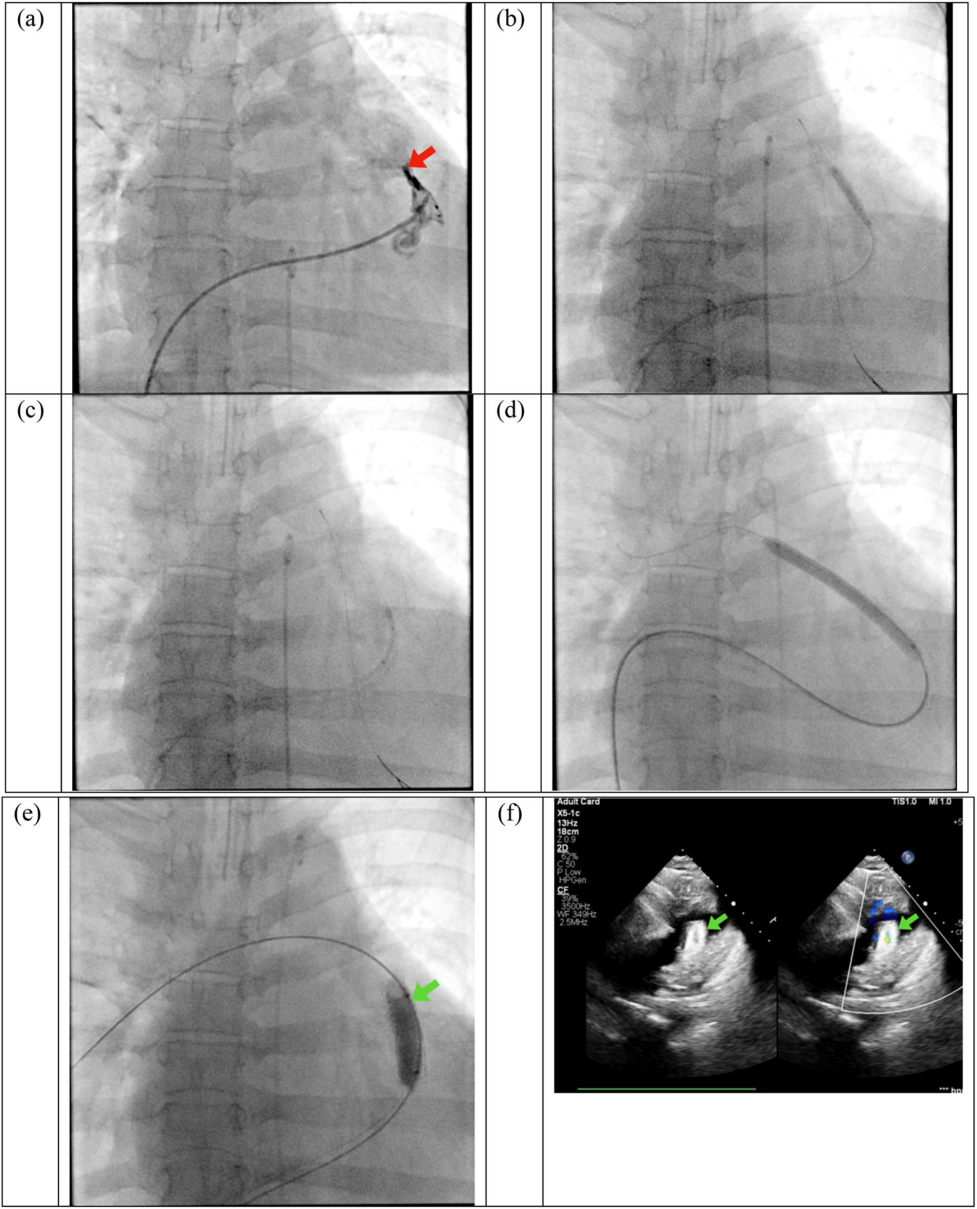
Right ventricular outflow tract (RVOT) stenting in the present patient. **(a)** Angiogram demonstrating severe RVOT stenosis with nearly absent pulmonary artery flow (*red arrow*). **(b)** Lesion predilation with a 4.0 × 18 mm Sapphire coronary balloon. **(c)** Balloon-assisted catheter advancement technique. **(d)** Inflation with a 6.0 × 60 mm Mustang vascular balloon. **(e)** Deployment of a 10.0 × 29 mm Omnilink vascular stent. **(f)** Post-procedural echocardiography confirming appropriate stent position (*green arrow*).

Preparation was carried out as per our standard protocol. Intraprocedural unfractionated heparin 700 IU was administered after vascular access. Even though the patient presented with severe hematemesis, with aortic saturation being critically low at 21%, the decision was made to proceed with the procedure. Baseline hemodynamic measurements showed right ventricular pressure of 189/62 mmHg and main pulmonary artery pressure of 126/87 mmHg. A baseline angiogram revealed a severely stenotic RVOT (Figure 1a). Lesion preparation using a 4.0 × 18 mm Sapphire coronary balloon was performed, inflated up to 12 atm nine times (Figure 1b). However, the guiding catheter could still not be advanced into the main pulmonary artery (MPA).

We then attempted a balloon-assisted catheter advancement technique using a 2.5 × 15 mm Sapphire coronary balloon (Figure 1c), but this was also unsuccessful. Subsequently, we used a 0.035-inch stiff soft hydrophilic wire, which allowed us to advance a 6.0 × 60 mm Boston Mustang balloon. This balloon was inflated up to 10 atm, also nine times (Figure 1d).

We then attempted to switch to a super stiff wire, but this maneuver was once again unsuccessful. Then, we tried to advance the 8.0 × 60 mm Mustang Vascular balloon using 0.035-inch soft hydrophilic super stiff wire, but still failed, as it was retracted to the right ventricle. Using the 0.035-inch soft hydrophilic stiff wire again, we advanced an 8.0 × 60 mm vascular balloon, which was inflated up to 10 atm ten times. The balloon was then pushed into the distal right pulmonary artery (RPA) and inflated in position after wire retraction.

At this stage, we inserted a 0.035-inch super stiff wire into the inflated balloon to prevent dislodgement—this was a key step and the technical highlight of this procedure. We then upsized to an 8.0 × 60 mm Mustang vascular balloon, inflating it up to 12 atm 19 times. Repeated balloon inflations were required because stent delivery was not performed through a long sheath as it was not available in our country; therefore, adequate lesion preparation was essential to allow safe stent advancement across the extremely tight and resistant infundibular obstruction. At this point, the oxygen saturation was already increased to 75%. Stent position was confirmed by repeated angiographic and TEE assessment before deployment. Following this step, a 10.0 × 39 mm Omnilink vascular stent was deployed and inflated to 12 atm. A second stent, 10.0 × 29 mm Omnilink vascular stent, was then placed and inflated up to 14 atm (Figure 1e). The stent diameter was selected to provide effective palliation of the infundibular obstruction while avoiding excessive oversizing relative to the 15-mm pulmonary annulus. The pulmonary valve was spared because the stents were positioned within the infundibular RVOT and did not compromise the pulmonary annulus. The result showed improved cardiac contraction and a significant increase in aortic saturation, rising to 92%. Post-stenting hemodynamic measurements showed a decrease in right ventricular pressure to 89/52 mmHg and main pulmonary artery pressure to 53/50 mmHg.

Post procedural echocardiography showed good stent shape and position (Figure 1f), no cramping, no pulmonary regurgitation, a right ventricular - pulmonary artery gradient of 43 mmHg, improvements in the right ventricular-pulmonary artery flow, a moderate tricuspid regurgitation with TVG 88 mmHg, and no pericardial effusion. Post-deployment echocardiography was used to confirm stent position, RVOT flow, pulmonary valve function, and absence of pericardial effusion.

**Figure 2.**
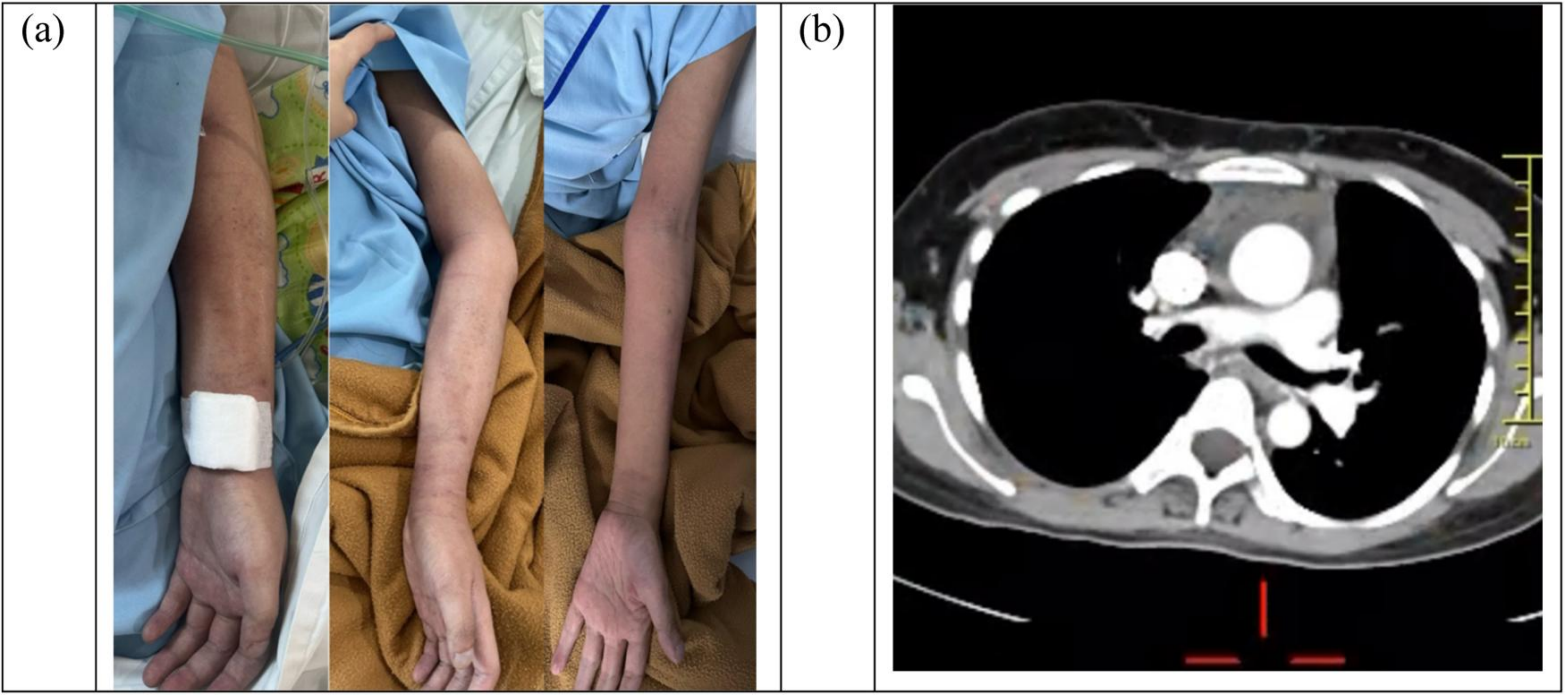
Post-procedural follow-up of the patient. **(a)** Serial clinical images of the left arm obtained on day 2 (left), day 4 (middle), and day 10 (right) after RVOT stenting. **(b)** Cardiac MSCT demonstrating confluent pulmonary arteries with a McGoon ratio of 2.21.

The patient was admitted to the CVCU and was extubated one day after the intervention. Her gastrointestinal bleeding resolved two days after the procedure. Post-procedural anticoagulation was withheld because of active gastrointestinal bleeding and severe thrombocytopenia. The DVT formed in her left arm gradually improved even though the patient was not given any anticoagulant (Figure 2a). Her platelet counts gradually increased following RVOT stenting and reached 87.000/uL 12 days after her admission, with her initial platelet count being 18.000/uL. As her clinical condition continued to improve, she was transferred to the intermediate ward, then to the general ward, and was eventually discharged.

The patient began phase 2 cardiac rehabilitation with an exercise program tailored to her 6-minute walk test results. The regimen was scheduled twice weekly for six weeks (a total of 12 sessions), aiming to achieve a target of over 6 METs. No adverse events were reported throughout the program. Notably, the patient showed an improvement in functional capacity, increasing from 2.9 METs to 4.8 METs.

Cardiac MSCT 2 months post procedural showed patent RVOT stent, with diameter of proximal RPA of 19.4 mm, distal RPA of 19.3 mm, diameter of proximal LPA of 13.0 mm, distal LPA of 14.7 mm, descending aorta diameter of 15.4 mm, the pulmonary artery was confluent with McGoon ratio of 2.21 (Figure 2b). At the latest follow-up, the patient's oxygen saturation was 92%, NYHA functional class was I-II, and left ventricular ejection fraction was 66% on echocardiography. Surgical conference concluded that definitive DORV repair with RVOT stent evacuation was feasible.

## Discussion

Adults with complex CHD is known to have a 4.36 higher risk of out-of-hospital cardiac arrest with a younger demography in comparison to those without (9, 10). In patients with RVOT obstruction, progressive right ventricular hypertrophy can exacerbate infundibular stenosis over time, and dynamic RVOT obstruction may trigger life-threatening tet spells or cardiac arrest even in adulthood, as observed in our patient (11, 12).

In adult patient with severe RVOT with hypoxia severe enough to eventually trigger cardiac arrest, RVOT stenting might be the most optimal lifesaving condition through increasing pulmonary blood flow. Our patient presented with profound hypoxia leading to cardiac arrest. Although total surgical repair is the definitive treatment for such lesions, it was not feasible in this emergency setting considering her comorbidities, including ongoing gastrointestinal bleeding, DVT, severe hypoxia (oxygen saturation of 21%), and a high-risk surgical profile. Given these factors, urgent RVOT stenting was the only plausible option to promptly restore pulmonary blood flow and stabilize the patient. Despite multiple procedural challenges related to the tight stenosis, two overlapping vascular stents were successfully deployed in the RVOT. RVOT stenting rapidly relieved right ventricular outflow obstruction, restoring antegrade pulmonary flow and lowering systemic venous pressure. This hemodynamic correction reduced venous congestion and stasis, the primary drivers of edema and thrombosis, while improved oxygenation alleviated hypoxia-induced endothelial dysfunction and procoagulant activity. As a result, gastrointestinal bleeding resolved, and the deep vein thrombosis improved despite withholding anticoagulation, indicating that the intervention itself acted as a systemic hemodynamic reset rather than merely a local repair.

The most common indication for urgent RVOT stenting is cyanotic spell (8) as seen in our patient. Although there are no formally established criteria to define urgent RVOT stenting, in cases of rapid clinical deterioration such as profound hypoxia or hemodynamic instability, intervention should be prioritized without delay. In this case, the patient's critical condition necessitated immediate action, as postponement would likely have led to irreversible organ damage or death.

RVOT stenting serves as a less invasive alternative to surgical shunts for patients unsuitable for immediate full repair. Indications for RVOT stenting in adults include profound desaturation (oxygen saturation <40%–50%), severely reduced left ventricular ejection fraction (LVEF <40%), high surgical risk for shunt placement, or in those with high operative risk for total correction even with adequate pulmonary artery size (13). In such patients, timely RVOT stenting may be life-saving and acts as a bridge to definitive surgery at a later stage (14).

Long-term complications must be considered, particularly in adults with larger cardiac structures, long-standing cyanosis, and abnormal RVOT geometry. Potential complications include stent fracture, migration, in-stent restenosis, stent thrombosis, endocarditis, pulmonary regurgitation if the valve is crossed, and access-site bleeding or thrombosis. In late-presenting Fallot-type physiology, the hyperdynamic and muscular RVOT may expose the stent to repetitive compressive forces and hinge motion, increasing the risk of stent deformation or fracture. Our previous experience demonstrated that double stenting may serve as a temporary strategy in patients with a fractured RVOT stent, helping to restore pulmonary blood flow and bridge the patient to definitive surgical repair, although it should not be considered a long-term solution (15). Therefore, serial clinical follow-up, echocardiography, and cross-sectional imaging are essential while awaiting definitive repair. In the present case, two overlapping stents were intentionally implanted to provide stronger radial support and more adequate scaffolding across the severely narrowed and dynamic RVOT. This strategy was chosen to maintain RVOT patency until the patient became suitable for definitive surgical correction.

Thrombosis in the RVOT stent may complicate cases particularly in patients with cyanotic congenital heart disease (CHD) who are at increased risk of thrombosis. This elevated risk may be attributed to prothrombotic conditions such as dilated, slow-flow cardiac chambers and vessels, as well as elevated hematocrit levels secondary to hypoxemia. Our patient had suffered from polycythemia and a dilated right ventricle, both of which may have contributed to a prothrombotic state. Additionally, DVT was detected in her upper extremities. On follow-up, the DVT had improved significantly following RVOT stenting. Hypoxia can activate pro-coagulation pathways, leading to increased tissue factor expression and impaired fibrinolysis. RVOT stenting enhances oxygen delivery and reduces hypoxemia, which may, in turn, decrease thrombogenicity (16). Echocardiography evaluation also did not show any thrombus in RVOT stent.

Our patient also had a high risk of bleeding due to a stress-related gastrointestinal ulcer, as well as active bleeding and thrombocytopenia at the time of intervention. Thrombocytopenia is commonly observed in cyanotic congenital heart disease (CCHD). In a study by Lill et al., 25% of patients with cyanotic CHD had platelet counts below 100 × 10⁹/L. The pathophysiological mechanisms responsible for thrombocytopenia in CCHD include a decreased platelet production, reduced megakaryocyte production, increased platelet destruction, and enhanced platelet activation. Megakaryocytes originate in the bone marrow and lodge in the pulmonary capillary bed, where the platelets are subsequently produced. In cyanotic CHD, right-to-left shunting reduces the number of megakaryocytes reaching the pulmonary bed, thereby decreasing platelet production (17). RVOT stenting increases pulmonary blood flow, which may explain the rise in platelet count following the procedure and the resolution of bleeding.

In the early post-procedural period, anticoagulation was withheld because the bleeding risk was considered prohibitive. During follow-up, the upper extremity DVT had clinically resolved without anticoagulation after improvement in oxygenation and systemic venous congestion following RVOT stenting. Therefore, routine long-term anticoagulation was not planned in this patient, particularly given her major bleeding risk. Instead, close clinical and imaging surveillance was emphasized. Definitive repair is planned after stabilization, with surgical conference recommending DORV repair and RVOT stent evacuation. The MSCT finding of confluent pulmonary arteries and a McGoon ratio of 2.21 supports the feasibility of complete repair after optimization.

## Conclusion

Urgent RVOT stenting can serve as a feasible therapeutic option in selected high-risk ACHD patients, providing effective restoration of pulmonary blood flow and oxygenation. This case also illustrates how tailored interventional strategies may contribute to the stabilization of both thrombotic and bleeding complications. Nevertheless, further clinical experience is needed to establish its broader applicability and long-term outcomes.

## Data Availability

The original contributions presented in the study are included in the article/Supplementary Material, further inquiries can be directed to the corresponding author.
